# Case Report: Ensartinib for gastric epithelioid inflammatory myofibrosarcoma with STRN-ALK fusion

**DOI:** 10.3389/fonc.2023.1252221

**Published:** 2023-10-05

**Authors:** XiaoQing Li, JingFan Zheng, XinYi Li, YuYu Chen, Kang Liu, FangChao Li, Zhong Lu

**Affiliations:** ^1^ School of Clinical Medicine, Weifang Medical University, Weifang, Shandong, China; ^2^ Department of Oncology, Affiliated Hospital Of Weifang Medical University, Weifang, Shandong, China

**Keywords:** inflammatory myofibroblastoma, STRN-ALK, ensartinib, epithelioid inflammatory myofibroblastic sarcoma, anaplastic lymphoma kinase

## Abstract

Epithelioid inflammatory myofibroblastic sarcoma (EIMS) is a highly aggressive malignant subtype of inflammatory myofibroblastoma (IMT) associated with poor prognosis. IMT can occur in various parts of the body, most frequently in the lungs, followed by the mesentery, omentum, retroperitoneum, and pelvis, among other areas; however, it is exceptionally rare in the stomach. Anaplastic lymphoma kinase (ALK) is a critical driver of lung cancer development and is currently the “gold standard” target for non-small cell lung cancer treatment. However, there are few reports on the use of ALK inhibitors for EIMS, necessitating further investigation. A male patient with postoperative inflammatory myofibroblastic sarcoma of the stomach received postoperative chemotherapy and had a stable outcome. However, a repeat CT scan performed 11 months later revealed disease progression. The patient later underwent immunohistochemistry testing that indicated ALK positivity, and next-generation sequencing revealed STRN-ALK fusion. Ensartinib 225 mg qd was administered as recommended, and the patient experienced only mild pruritus and no adverse effects such as rash. Eight months after CT follow-up, the patient’s subseptal soft tissue nodules had decreased, and the outcome was assessed as a partial response. The findings of this case report introduce a novel strategy for treating ALK-positive EIMS that utilizes ensartinib, a drug with previously demonstrated success in the treatment of ALK-positive cancer.

## 1 Introduction

Epithelioid inflammatory myofibroblast sarcoma (EIMS) is a rare malignant subtype of inflammatory myofibroblastoma (IMT), which was first proposed and named by Marino-Enriquez et al. (1). It can manifest at any stage of life, but commonly occurs in adults, and exhibits a noticeable male preponderance (2). IMT are primarily composed of myofibroblastic spindle cells and are often accompanied by plasma cell or lymphocytic infiltration (3). In contrast, the inflammatory cells present in the interstitium of EIMS are predominantly neutrophils (4). Not only do the two differ histologically, but they also differ in terms of their clinical presentation. EIMS is highly aggressive, has a high recurrence rate, and can progress rapidly, leading to mortality (1). The presence of the characteristic RANBP2-ALK (5) gene fusion in EIMS often signifies a poor prognosis. However, the STRN-ALK fusion is infrequently observed in cases of EIMS.

Ensartinib is a novel, second-generation ALK tyrosine kinase inhibitor (TKI) with improved efficacy against central nervous system (CNS) metastases (6). In contrast to other ALK TKIs, ensartinib is effective against certain ALK mutations (e.g., L1196M and C1156Y) and other sites of action (7).

## 2 Case description

**Figure 1:**
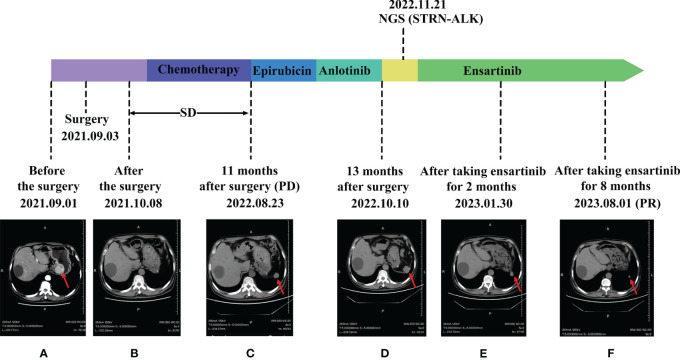
Comparison of images before and after ensartinib treatment. **(A)** Before surgery; **(B)** After surgery; **(C)** Postoperative review in November; **(D)** anlotinib post-treatment; **(E)** After 2 months on Ensartinib; **(F)** After 8 months on Ensartinib.

**Figure 2:**
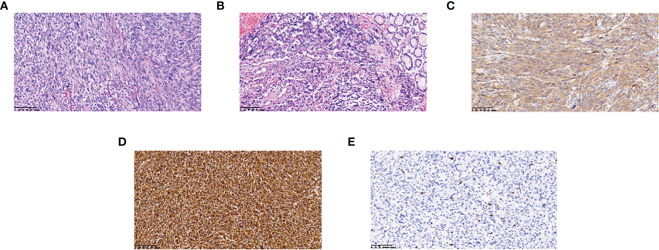
Hematoxylin-eosin staining and immunohistochemistry findings: **(A)** The lesion is infiltrated by epithelioid cells and numerous mature inflammatory cells (magnification, x200); **(B)** Tumor tissue junction with normal tissue (magnification, x200); **(C)** ALK positivity (magnification, x200); **(D)** CD117 weakly positive (magnification, x200); **(E)** Vimentin positivity (magnification, x200).

An middle-aged Asian male patient was admitted to our hospital with hematochezia for 4 days. The color of stools was black red, and the patient had no other symptoms such as abdominal pain. He was in good general condition, and his cardiopulmonary examination showed no obvious abnormalities. And the routine blood test results and the main indexes of liver and kidney function were basically normal. A computed tomography (CT) scan of the abdomen revealed a large mesenchymal tumor located at the base of the stomach (**Figure 1A**), while gastroscopic examination identified a bleeding, ruptured gastric mass. Histopathological examination revealed a possible pseudosarcomatous response with histiocytic hyperplasia. On September 1, 2021, the patient underwent laparoscopic partial gastrectomy under general anesthesia with tracheal intubation, resulting in complete removal of the gastric mass (**Figure 1B**). Postoperative pathological results indicated a mesenchymal tumor with a fragmented mass and a total volume of 10 × 8 cm × 2.5 cm. Immunohistochemistry showed that the tumor was Vimentin (+), CD68 (+), ALK (+), CD99 (+), SDHB (+), S-100 (partially +), SAM (slightly +), EMA (weakly +), CD34 (vascularly +), CD117 (sporadically +), Dog-1 (-), Desmin (-), HMB45 (-), CK wide (-), and had a Ki-67 index of 20%. Based on the morphological characteristics from microscopic pathology and immunohistochemistry, a diagnosis of EIMS was made. The morphological diagnosis in this case was primarily based on the following criteria: 1) microscopic tumor cells displaying an epithelioid morphology with prominent nucleoli and easily discernible nuclear divisions were observed (**Figures 2A, B**); 2) interstitial infiltration with a large number of mature inflammatory cells, including neutrophils, lymphocytes, and eosinophils, and mucous degeneration were observed in the interstitium (**Figures 2A, B**); and 3) robust positivity for ALK (**Figure 2C**) was detected in plasma samples. Diagnosis based on immunohistochemical results necessitates differential diagnosis between gastrointestinal mesenchymal tumors, malignant melanoma, and other tumors with an epithelioid phenotype. In this case, ALK positivity was specific to this tumor; the presence of CD117 (scattered +) (**Figure 2D**) and absence of Dog1 (-) ruled out mesenchymal tumors, the absence of HMB45 (-) ruled out malignant melanoma, and diffuse positivity for vimentin (**Figure 2E**) was consistent with EIMS.

Following surgical intervention, the patient received five cycles of paclitaxel in combination with cisplatin and a subsequent four cycles of maintenance chemotherapy with single-agent nedaplatin. During treatment, the efficacy was assessed as stable disease (SD). Upon a post-surgery review in November, an increase in the size of the left subphrenic soft tissue nodule was noted, indicating progression (PD) (**Figure 1C**). In response, the treatment regimen was altered to include intravenous chemotherapy with single-agent epirubicin. and the patient subsequently underwent two rounds of targeted therapy with Anlotinib, Despite this, the disease continued to progress (**Figure 1D**).

**Figure 3:**
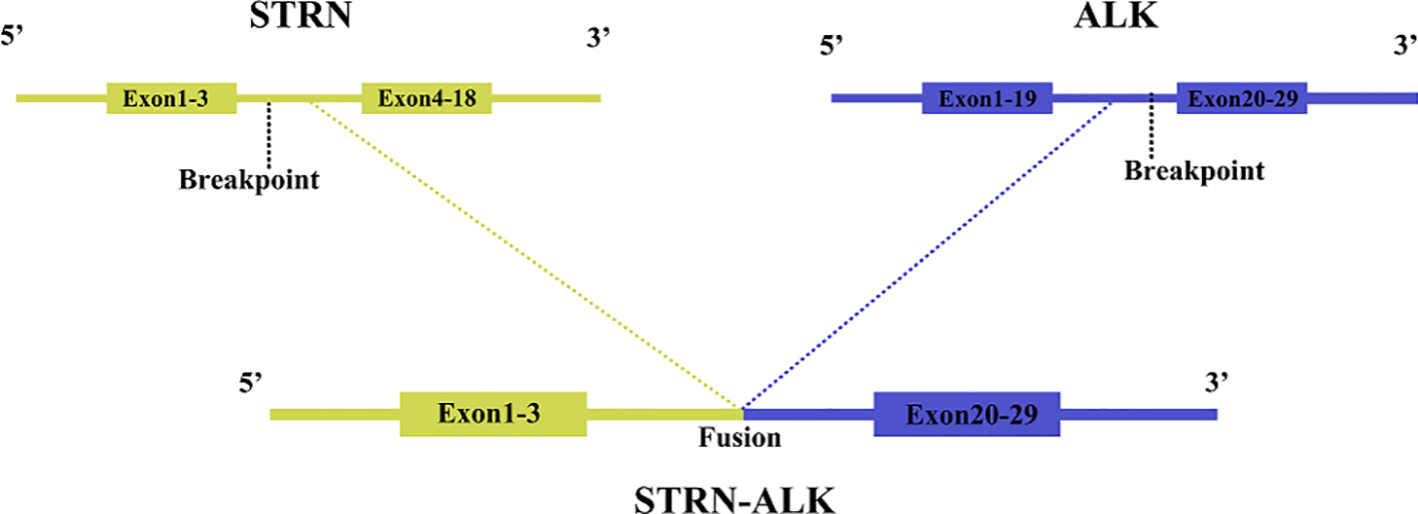
The schematic structure of the STRN-ALK fusion points.

One year and two months after surgery, the patient underwent genetic testing with high-throughput sequencing (104 genomes, shihe, china) using a biopsy sample from the abdominal mass. Testing identified a STRN-ALK fusion with a mutation abundance of 32.31%, and a KIT mutation with a mutation abundance of 1.24%. The STRN-ALK transcript was found to be located in exon 3 of STRN and in exon 20 of ALK (**Figure 3**). The patient was subsequently treated with ensartinib 225 mg once daily and monitored using CT follow-up. After two months of ensartinib treatment, a notable reduction in the subseptal soft tissue nodules was observed (**Figure 1E**). Eight months later, CT scans showed a significant reduction in the number of subdiaphragmatic soft tissue nodules, and the efficacy was evaluated as a partial response (PR) according to the first edition of the Criteria for the Evaluation of the Efficacy of Solid Tumours (**Figure 1F**). Mild pruritus emerged after two months of ensartinib treatment. The patient is currently stable and undergoing regular outpatient monitoring while receiving ensartinib treatment.

## 3 Discussion

IMT is a rare mesenchymal-derived tumor with a potential for recurrence (8). In roughly 50% of IMT patients, there is immunostaining for ALK in either a membranous or perinuclear pattern (9). EIMS is an aggressive variant of IMT that primarily comprises epithelioid cells with round, short-spindle, polygonal, and oval morphologies. A large infiltration of inflammatory cells dominates the interstitium, with minor infiltration of plasma cells, lymphocytes, and eosinophils (4). Yu et al. summarized the sites of EIMS as mainly located in the abdominal cavity, including the mesentery and omentum, occasionally in the liver, lungs, and other organs, and rarely in the stomach (10). The primary clinical manifestations are abdominal pain or masses, occasionally accompanied by ascites (10). Immunohistochemistry mainly displays the expression of ALK and desmin, with ALK being characteristically membrane- or perinuclear-positive, though sometimes exhibiting cytoplasmic staining with perinuclear hollowing. The expression of EMA, CD3, CD20, CD117, and Dog-1, among others, is usually absent or rare (11). EIMS is a rare condition, and its pathomorphology often leads to confusion with other tumors. Hence, identifying ALK rearrangements using FISH, PCR, or gene sequencing can aid in the accurate diagnosis of EIMS, and consequently in developing the correct treatment plan.

ALK was initially cloned in 1994 as a fusion protein in mesenchymal lymphoma (ALCL) (12). ALK fusion proteins autophosphorylate, resulting in constitutively active tyrosine kinases that activate downstream cell signaling pathways, such as RAS/ERK, PI3K/Akt, and JAK/STAT, ultimately leading to cancer (13). The most common ALK fusion protein in patients with ALCL is NPM1-A, accounting for 70-80% of cases (13). In IMT, ALK can be fused with TPM3, TPM4, CLTC, ATIC, and other genes (14–16), and the different fusion partners of ALK lead to three different ALK staining patterns: nuclear membrane staining, granular cytoplasmic staining, and smooth cytoplasmic staining (17). Recently, a new recurrent RRBP1-ALK fusion gene was identified, which retains the coiled helix structure of N-terminal RRBP1, leading to the activation of the ALK oncogenic mechanism (18). The RANBP2-ALK fusion protein in IMT promotes cell proliferation (19) and has a unique nuclear membrane localization. RRBP1-ALK and RANBP2-ALK are the only two fusion genes that have been shown to cause recurrent oncogenesis in EIMS (19), making the detection of the RANBP2-ALK fusion protein of great diagnostic and prognostic value in EMIS. Mesenchymal large cell lymphoma has significant clinical, immunophenotypic, and morphological overlap with EIMS, but the RANBP2-ALK gene has not been reported in mesenchymal large cell lymphoma; therefore, RANBP2-ALK gene fusion measured by RT-PCR can differentiate EIMS from mesenchymal large cell lymphoma (20). Fusion of the chaperone striatum protein (STRN) gene with ALK has been reported in thyroid cancer, where it is the most common form of ALK fusion (21). A mouse model of thyroid-specific STRN-ALK fusion expression has been shown to drive the development of hypofractionated thyroid cancer (PTDC) (22). The coiled helical structural domain retained in the STRN-ALK fusion protein is essential for the activation of ALK tyrosine kinase (21), and translocation induces MAPK activation, increasing cell proliferation and transformation (23), leading to cancer. In the present case study, the ALK gene rearrangement was detected using the more sensitive molecular technique of gene sequencing, allowing the STRN-ALK transcript to be identified as consisting of a fusion between exon 3 of STRN and exon 20 of ALK. Since the kinase region of ALK is located after exon 20, and previous reports have shown that STRN-ALK drives the development of thyroid and renal cancers (24), we speculate that the STRN-ALK gene fusion plays a vital role in promoting cancer development in this case and contributes to the high value-added state and particular epithelioid morphology of EIMS. In lung adenocarcinoma, the most common ALK fusion is the EML-4 fusion (25); however, since the STRN-ALK fusion gene was first reported in 2013 (26), the presence of STRN-ALK fusions in thyroid and lung adenocarcinomas has been well documented. To date, no STRN-ALK fusions have been reported in gastric epithelioid inflammatory myofibroblastoma.

Currently, there is no established standard of care for patients with EIMS. Clinical experience suggests that surgical resection is the preferred treatment for inflammatory myofibroblastic sarcomas, regardless of tumor location. However, the efficacy of postoperative adjuvant chemotherapy and immunotherapy remains unclear. EIMS has a higher mortality rate and a greater propensity for early recurrence and metastasis following surgery than typical IMT. Abdominal EIMS is particularly prone to progression, with metastatic sites typically involving the liver, lungs, and small intestine (4). In some patients with ALK rearrangements, remarkable success has been achieved by incorporating ALK inhibitors as part of their treatment; in our case, the identification of the ALK rearrangement was delayed due to patient-related reasons. Postoperative chemotherapy is ineffective in controlling disease progression. The central nervous system is the most common site of disease progression after treatment with crizotinib, this is the advantage of second-generation ALK inhibitors over first-generation (27). Therefore, our choice of the subsequent treatment regimen was based on two primary factors: 1) better efficacy against STRN-ALK fusion mutations, and 2) prevention of CNS metastases and control of disease progression. Crizotinib, an ALK inhibitor, has been approved as the first-line treatment because of its significantly prolonged median progression-free survival compared to chemotherapy (10.9 vs. 7.0 months) (21). Sasaki et al. reported the development of crizotinib resistance in patients with RANBP2-ALK-positive EIMS (28); the mechanisms of resistance may involve secondary ALK mutations, ALK amplification, and activation of bypass signaling networks. The CNS is the most common site of disease progression following crizotinib treatment. Therefore, the search for novel and efficacious treatments is imperative to address the problem of acquired resistance and poor CNS efficacy. In recent years, newer generation ALK inhibitors, including ensartinib, ceritinib, aletinib, and bucatinib, have been introduced. Compared to crizotinib, the smaller molecular structure of ensartinib increases its lipid solubility and affinity to facilitate blood-brain barrier penetration. Preclinical data reveals ensartinib to be more effective compared to other second-generation ALK-1 drugs including crizotinib (29). One trial tested the penetration of crizotinib versus ensartinib through the BBB in mice and showed that ensartinib was more effective against ALK-positive brain metastases (30). Ensartinib demonstrated strong antitumor activity in H3122 lung cancer xenografts, with IC50 values of 0.015 and 0.180 mol/L for ensartinib and crizotinib, respectively, more than 10 times the efficacy of crizotinib (30). A phase I-II study showed that ensartinib inhibited mutant sites such as F1174 and C1156Y and had strong inhibitory effects on targets such as ROSE1, TRK, and MET, in addition to ALK (31). Given the additional target coverage and greater cranial penetration, we speculate that patients could benefit directly from second-generation ensartinib therapy. Based on previous case reports, ensartinib has demonstrated better efficacy in the treatment of brain metastatic non-small cell lung cancer with STRN-ALK fusion (32). In the treatment of epithelioid inflammatory myofibroblastoma with RANBP 2-ALK fusion with ensartinib, a PR was observed in patients after 4 months, and CT scans showed partial tumor shrinkage (33). An article reported the effective treatment of a GCC 2-ALK fusion case with ensartinib, in which the patient maintained PR after 7 months of follow-up and remained in progression-free survival after 11 months of follow-up (34). A case report of effective treatment with ensartinib in a patient carrying the acquired resistance mutation ALk l1171N lung adenocarcinoma, It successfully solved the problem of ALK resistance (35). Previous case reports have shown that ensartinib is effective for the direct treatment of epithelioid inflammatory myofibroblastic sarcoma. Compared with other second-generation ALK-TKI drugs, ensartinib is more cost-effective. Therefore, we used ensartinib to treat EIMS with STRN-ALK fusions. A phase I-II human clinical trial in the United States recommended a dose of ensartinib of 225 mg for 28 days. When the dose was increased to 250 mg, adverse reactions such as a grade 3 rash were observed, while the drug activity of ensartinib did not change. It has also been shown that food has no significant effect on the absorption of Ensartinib (31). Adverse drug reactions were observed in 86% of patients who underwent treatment with an ALK-TKI (31). If patients experience serious adverse reactions such as rash during treatment, the dose administered should be reduced as appropriate. In this case, the patient experienced mild pruritus in the second month and did not experience any adverse reactions, such as rash. The patient is still on ensartinib 225 mg qd and has been on it for over 9 months with no discomfort other than mild pruritus symptoms. In this case, a 1.24% mutation in KIT was identified using NGS. KIT is a type III receptor tyrosine kinase, is involved in a variety of signaling pathways, including the PI 3 K pathway and mitogen-activated protein kinase (MAPK) pathways, and is responsible for cell growth and proliferation (36). It has been shown to be a driver mutation in a variety of cancers, such as gastrointestinal mesenchymal tumors (37), and has also been shown to be a possible new molecular target in acute myeloid leukemia (38). However, the specific mutational significance in this case is unknown.

## 4 Conclusion

To summarize, we present the case of a patient who underwent surgery for gastric inflammatory myofibroblastoma that was staged as PT2NOMO stage IIIA, in accordance with the CSCO guidelines. To the best of our knowledge, this is the first reported case of the successful treatment of gastric epithelioid inflammatory myofibroblast sarcoma with STRN-ALK fusion, suggesting a novel approach for patients with EIMS with this fusion type. However, the limited number of studies investigating the efficacy of ensartinib in patients with STRN-ALK fusion highlights the need for further patient follow-up and analysis of additional clinical data.

## Data availability statement

The original contributions presented in the study are included in the article/supplementary material. Further inquiries can be directed to the corresponding author.

## Ethics statement

This study was approved by the ethic committee of Medical Research Ethics Committee of Affiliated Hospital of Weifang Medical University. All procedures performed in studies involving human participants were in accordance with the ethical standards of the institutional and/or national research committee and with the 1964 Helsinki declaration and its later amendments or comparable ethical standards. Written informed consent was obtained from the patients for the publication of this case report.

## Author contributions

XQL was responsible for editing the main manuscript text, JFZ, XYL, YYC and FCL were responsible for writing the pictures, ZL and KL were mainly responsible for reviewing the manuscript, and all the authors participated in reviewing the manuscript. All authors contributed to the article and approved the submitted version.
